# Bacteriophage-encoded lethal membrane disruptors: Advances in understanding and potential applications

**DOI:** 10.3389/fmicb.2022.1044143

**Published:** 2022-10-26

**Authors:** Gayan S. Abeysekera, Michael J. Love, Sarah H. Manners, Craig Billington, Renwick C. J. Dobson

**Affiliations:** ^1^Biomolecular Interaction Centre and School of Biological Sciences, University of Canterbury, Christchurch, New Zealand; ^2^Health and Environment Group, Institute of Environmental Science and Research, Christchurch, New Zealand; ^3^Department of Biochemistry and Molecular Biology, University of Melbourne, Melbourne, VIC, Australia

**Keywords:** bacteriophage, holins spanins, membrane protein, holins spanins applications, holins spanins future potential potential, alphaFold structure

## Abstract

Holins and spanins are bacteriophage-encoded membrane proteins that control bacterial cell lysis in the final stage of the bacteriophage reproductive cycle. Due to their efficient mechanisms for lethal membrane disruption, these proteins are gaining interest in many fields, including the medical, food, biotechnological, and pharmaceutical fields. However, investigating these lethal proteins is challenging due to their toxicity in bacterial expression systems and the resultant low protein yields have hindered their analysis compared to other cell lytic proteins. Therefore, the structural and dynamic properties of holins and spanins in their native environment are not well-understood. In this article we describe recent advances in the classification, purification, and analysis of holin and spanin proteins, which are beginning to overcome the technical barriers to understanding these lethal membrane disrupting proteins, and through this, unlock many potential biotechnological applications.

## Introduction

Bacteriophage are a diverse group of viruses that obligately infect bacteria and are ubiquitously found in nature (Suttle, 2005). They are microorganisms of growing scientific interest with, as of July 2022, 4,163 complete bacteriophage genome entries in the National Center for Biotechnology Information (NCBI; Brister et al., 2015). As parasitic organisms co-evolved alongside bacteria, bacteriophages are equipped with specialised bacterial infection mechanisms and novel biomolecules with potential for application as therapeutics or in industrial processes (Roucourt and Lavigne, 2009; Hampton et al., 2020).

Bacteriophage encoded cell lysis proteins are one such group of novel biomolecules that comprise endolysins, holins and spanins. The discovery of endolysin activity dates back to 1957, when Jacob et al. reported that endolysins effectively kill bacteria (Jacob et al., 1957; Jacob and Fuerst, 1958). The endolysin was found to be encoded by the R gene of bacteriophage lambda (Bieŉkowska-Szewczyk et al., 1981). Subsequently, a nonsense mutation in the lysis cassettes of bacteriophages T4 and lambda led to the identification of another lysis protein, holin, encoded by the lambda S gene (Josslin, 1970; Josslin, 1971; Reader and Siminovitch, 1971). This discovery transformed our understanding of endolysin-mediated cell lysis in bacteriophages by revealing its multifactorial nature. Later, the discovery of spanins, encoded by the Rz-Rz1 genes in lambda, provided another key protein of bacteriophage-mediated cell lysis (Young et al., 1979). Holins and spanins were later identified as transmembrane proteins that accumulate in the membrane to disrupt the inner membrane and outer membrane of the bacteria (Reader and Siminovitch, 1971; Young et al., 1979; Wilson, 1982), whereas endolysins degrade the peptidoglycan layer of the cell wall (Bieŉkowska-Szewczyk et al., 1981). Together, these proteins help to cleave highly conserved bonds of essential components of the peptidoglycan, inner membrane, and outer membrane (Nelson et al., 2012). Thus, endolysins, holins, and spanins have gained increasing research interest over the past few decades. However, unlike endolysins, attempts to produce recombinant holins and spanins using *Escherichia coli* expression systems have proven difficult due to their toxicity in the bacterial cell expression system (Pang et al., 2009; Lu et al., 2020). As such, holins and spanins remain poorly studied in comparison to endolysins (Pang et al., 2009).

In this review, we present the latest developments in the understanding of holin and spanin membrane disrupting proteins, including the mechanisms of action, potential applications, and the challenges to be overcome for their future application in bacterial therapy and other industrial uses.

## Holin classification and mechanism

Holins have two characteristic features: (1) they can be triggered to form a pore by the uncoupler dinitrophenol to initiate premature membrane disruption, and (2) their structure contains at least one transmembrane α-helical segment (Garrett et al., 1981; Garrett and Young, 1982; Gründling et al., 2001; Reddy and Saier, 2013). In addition, holins and endolysins isolated from heterologous bacteriophages often have interchangeable activity (Rennell and Poteete, 1985; Young, 1992). Interestingly, holin-like proteins are also found in virus-free mammalian cells and bacteria, playing key functional roles such as programmed cell death (e.g., the Bak, Bax, CidA, LrgA proteins; Patton et al., 2005; Pang et al., 2011), biofilm formation (e.g., the CidA, LrgA proteins; Sharma-Kuinkel et al., 2009; Moormeier et al., 2013), and gene transfer (e.g., GTA holin; Lang et al., 2012).

**Figure 1:**
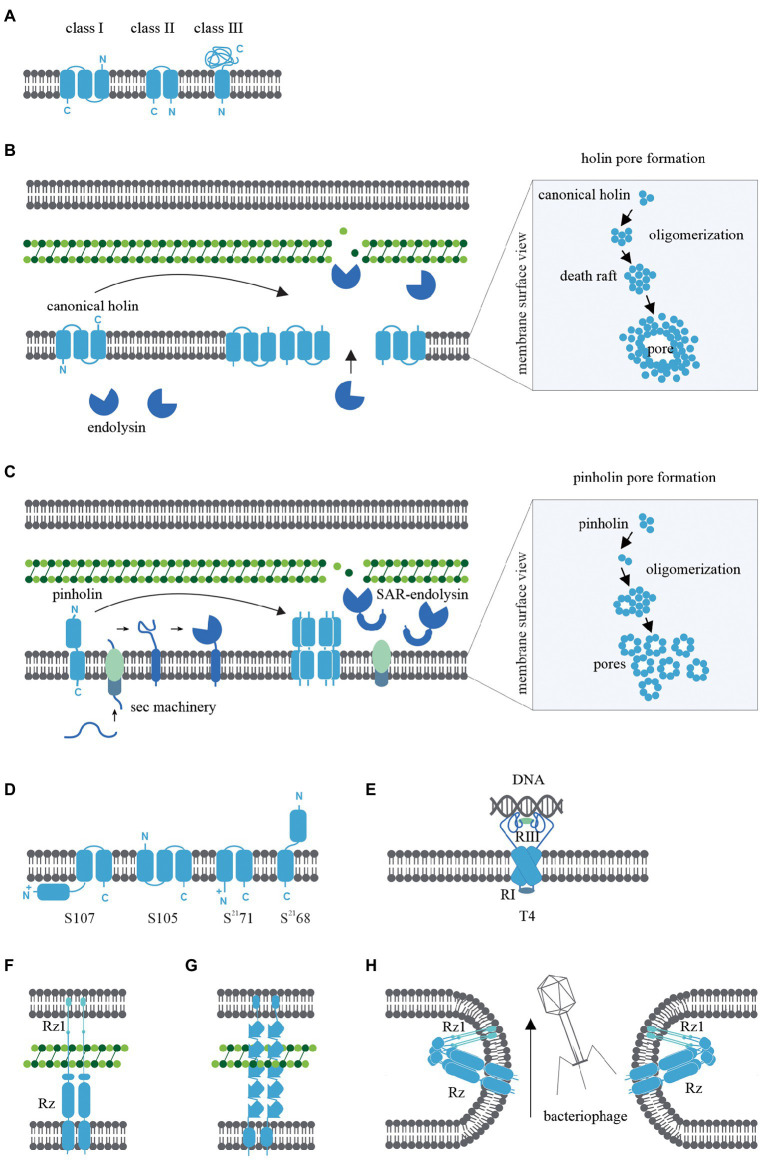
**(A)** Transmembrane α-helical segment topology of the most studied holin classes (I-III). **(B)** Schematic representation of canonical pathway: Following the late gene expression initiated in the bacteriophage lytic cycle, canonical holins and endolysins accumulate in the inner membrane and cytoplasm, respectively. At an allele-specific time, holins make micron-scale holes in the inner membrane and endolysins escape to the periplasm, degrading the peptidoglycan. **(C)** Schematic representation of pinholin pathway: When late gene expression is initiated, pinholins and inactive SAR endolysins accumulated in the inner membrane. At an allele-specific time, pinholins make heptameric channels with a lumen of ~2 nm which destabilised the proton motive pump. SAR endolysins transform into their active form as they are sensitive to the proton motive pump and finally degrade the peptidoglycan. **(D)** Topology of lambda and lambdoid bacteriophage ϕ21 holins (S105, S^21^68) and its antiholins (S107, S^21^71). **(E)** T4 holin complex can make a complex with its RI, RIII antiholins, and DNA to respond to superinfections. **(F)** Two-component spanins **(G)** Unimolecular spanins **(H)** Fusion inner and outer membrane.

Bacteriophage holins are classified according to the topology of the transmembrane α-helical segments and three classes have been widely studied. Class I holins have three transmembrane α-helical segments arranged N-out and C-in configuration (Smith et al., 1998), whereas Class II holins consist of two transmembrane α-helical segments arranged N-in and C-in (Figure 1A; Smith et al., 1998). Examples include the class I holin S105 of lambda and the class II holin S^21^68 of lambdoid bacteriophage ϕ21 (Smith et al., 1998). These two classes of holins are the most abundant holins described in bacteriophages so far. Class III holins have one transmembrane α-helical segment and a large periplasmic domain arranged N-in and C-out (Figure 1A) and this class of holin is found in T4-like and T5-like phages (Shi et al., 2012). An *in silico* study of 52 holin families using the transporter classification database showed that the maximum number of transmembrane α-helical segments harboured by a holin protein is four (Reddy and Saier, 2013); thus, there could potentially be eight different topologies of holins in nature.

The primary function of bacteriophage holins is to initiate the formation of inner membrane pores during the lytic cycle through a process called ‘triggering’ (Chang et al., 1995). Triggering provides the cytoplasmic endolysins access to the peptidoglycan layer and subsequent degradation. Holins control the timing of cell lysis (the ‘lysis clock’). The lysis clock is regulated by factors including the allele type (Chang et al., 1995), rate of transcription or translation of the holin gene (Singh and Dennehy, 2014), antiholins (discussed later in the review), and environmental conditions (e.g., the lysis clock of T4 holin; Wang et al., 1996; Gründling et al., 2000; Tran et al., 2005). Studies of the lambda S105 and S^21^68 holins in the last few decades have focused on elucidating the triggering pathway based on microscopic, biomolecular, functional, and structural studies (Chang et al., 1995; Dewey et al., 2010; White et al., 2011; Pang et al., 2013).

Two pathways have been proposed for triggering holin pore formation; the canonical and pinholin pathways. Each has distinctive morphologies of membrane pore formation and cell lysis.

### Canonical holins

The canonical pathway is illustrated in Figure 1B and is based on studies of the lambda S105 holin as a model for canonical holins. Using green fluorescent protein fusions (White et al., 2011), cryo-electron microscopy (Dewey et al., 2010; White et al., 2011), and cysteine-scanning accessibility studies (Savva et al., 2008; To and Young, 2014), a raft formation model has been proposed to describe the canonical holin pathway (Wang et al., 2003). Once late gene expression of the bacteriophage lytic cycle is initiated, homodimers of canonical holins accumulate in the inner membrane and endolysins amass in the cytoplasm. Accumulated canonical holins are labile and coalesce to form a two-dimensional structure in the inner membrane called a “death raft” (Wang et al., 2003). After ~1,000–3,000 holins accumulate in the raft, tight packing of the holins causes localised lipid depletion. The death rafts are permeable to ions and protons and compromise the integrity of the inner membrane. Leaching ions and protons across the death raft induces a local reduction in the proton motive force (Zagotta and Wilson, 1990; Chang et al., 1995; Savva et al., 2014). Further raft coalescence and decreasing proton motive force then lead to the sudden formation of a micron-scale hole (Figure 1B; Savva et al., 2014). This is a non-specific formation, as replacement cross-complement activity of holin proteins from other bacteriophages is observed (Young, 1992). Surprisingly, it has been shown that the infected bacterial cells remain viable until the hole is formed (Gründling et al., 2001). When the canonical holins form the micron-scale hole, preformed endolysins from the cytoplasm can escape to the periplasm and degrade the peptidoglycan layer (Reader and Siminovitch, 1971; Wilson, 1982).

### Pinholins

The S^21^68 holin gene from lambdoid bacteriophage ϕ21 was found to be incapable of complementing the S allele (S105) in lambda (Gründling et al., 2001) and this observation led to the discovery of the non-canonical pinholin pathway (Park et al., 2007; Figure 1C). S^21^68-GFP fusions and cysteine-accessibility experiments indicated that pinholins make heptameric channels (Pang et al., 2009) with a lumen of ~2 nm (Figure 1C) instead of the micron-scale holes induced by canonical holins (Savva et al., 2014). In the pinholin pathway, it appears that around 10^3^ heptameric channels evenly accumulate in the inner membrane to collapse the proton motive force (Pang et al., 2009, 2013).

Pinholins are typically found in bacteriophages that also encode signal-anchor-release type endolysins, with the signal sequence of the signal-anchor-release endolysin exploiting the host sec machinery. Unlike canonical endolysins, which accumulate in the cytoplasm in active form, signal-anchor-release endolysins accumulate in the inner membrane in an inactive membrane-tethered form. The tethered signal-anchor-release endolysins are proton motive force sensitive. When pinholins trigger channel formation, the proton motive force collapses and signal-anchor-release endolysins are released from the membrane to the periplasm where they refold into an active form and hydrolyse the peptidoglycan (Xu et al., 2005; Park et al., 2007). signal-anchor-release endolysins hydrolyse the peptidoglycan MurNac-GlcNac glycosidic bond. Therefore signal-anchor-release endolysins are members of the lysozyme-like superfamily and share a common “classic lysozyme” fold (Kuty, 2011; Oliveira et al., 2013).

### Antiholins

The S105 and S^21^68 holins have a dual-start motif in the upstream region of each coding gene that can also translate the S107 and S^21^71 proteins, respectively (Bläsi et al., 1990; Barenboim et al., 1999). These alternatively transcribed proteins are antiholins and are a key determinant in regulating lysis triggering time. The S107 and S^21^71 antiholins possess an additional positively charged residue in the N-terminus, which changes the structural topology compared to the functional holin form (Park et al., 2006; Figure 1D). During holin accumulation in the inner membrane, lipid depletion causes a proton motive force reduction leading to the conversion of antiholins to their functional form, thus removing the topological barrier and causing a sudden amplification of the functional holins. This, in turn, leads to a sudden reduction of proton motive force reduction and lytic cascade (49, 50). Although S107 and S^21^71 antiholins have been widely studied (White et al., 2010), the architecture of antiholins can be complex. For example, T4 phage harbour both cytoplasmic and periplasmic antiholins, which create a complex that can respond to periplasmic DNA resulting from superinfections that triggers a delay hole formation (Figure 1E; Krieger et al., 2020).

### Spanins

Bacteriophage spanins are unique to phage that infect Gram-negative bacteria, given that Gram-positive organisms do not possess an outer membrane. Spanins have a diverse genetic architecture, with two main types identified: two-component spanins (Figure 1F) and unimolecular spanins (u-spanins; Figure 1G). Recent *in silico* studies identified 528 two-component spanins and 58 unimolecular spanins from the NCBI reference sequence database (Berry et al., 2013; Cahill et al., 2017a; Kongari et al., 2018). Two-component spanins comprise two membrane proteins, an outer membrane lipoprotein (o-spanin) and an integral inner membrane protein (i-spanin). There are three different ways of encoding o-spanin and i-spanin in the phage genome. (i) two genes encoding these proteins may be nested (e.g., in lambda and T7 the o-spanin gene is nested within the i-spanin gene; Kongari et al., 2018), (ii) two genes may be overlapped (e.g., in P2 the o-spanin gene extends beyond i-spanin gene; Kongari et al., 2018), or (iii) two genes may be separated (e.g., in T4; Kongari et al., 2018). However, u-spanins (e.g., in T1) are encoded as a single gene in the bacteriophage lysis cassette (Summer et al., 2007).

The mechanism of spanin activity has been studied by examining mutations in the two-component spanin genes of lambda, which caused spherical deformed bacterial cells, indicating incomplete cell rupture (Summer et al., 2007). Furthermore, phase-contrast microscopy revealed the inner membrane and outer membrane were intact, but the peptidoglycan was not visible. Thus, the key role of spanins is theorised to be the fusion of the inner and outer membranes after peptidoglycan hydrolysis by endolysins, which leads to cell rupture (Berry et al., 2012). This membrane fusion model is supported by experiments that indicate spanins accumulate in the envelope as dimers bridging inner membrane and outer membrane (Berry et al., 2012, 2013). These bridges span the whole periplasm and are threaded through the peptidoglycan. Peptidoglycan layer avoids the formation of the spanin’s innate hairpin-like conformation. When endolysins begin to degrade the peptidoglycan, spanins oligomerize by coiled-coil periplasmic domains and fuse the inner membrane and outer membrane to release cytoplasm content to the environment (Figure 1H; Berry et al., 2010; Rajaure et al., 2015; Cahill et al., 2017a, 2017b).

Fluorescence microscopy and genetic studies suggest that u-spanins use similar accumulation and fusion mechanisms as two-component spanins, but rely on β-sheet oligomerization and single-molecule expansion through peptidoglycan (Kongari et al., 2018).

## Strategies for elucidating holin and spanin structure and function

Expression of holins and spanins under laboratory conditions is challenging due to their cellular toxicity. Many studies have observed that the viability of bacterial cells decreased rapidly after the induction of plasmids containing holin and spanin genes (Chang et al., 1995; Summer et al., 2007; Lu et al., 2020). Despite the low viability of the resultant bacterial cells, most of the studies have investigated holins and spanins utilising His+SUMO / His tagging and solubilization in non-ionic detergent (n-Dodecyl-B-D-maltoside; Park et al., 2006; Savva et al., 2008; Pang et al., 2009). This has enabled progress in characterising some of the topology and function of holins and spanins using site-directed DNA mutagenesis, chemical cross-linking, and cysteine modification analysis (Savva et al., 2008; Pang et al., 2009; Berry et al., 2013). The use of a planar lipid bilayer system in place of detergents has also been suggested (Young, 2013). However, the low yield of holins and spanins produced by these techniques has hindered high-resolution structural studies such as crystallography or NMR (Pang et al., 2009).

Recently the first biophysical study was conducted on the S^21^68 pinholin, whereby S^21^68 and its inactive version (S^21^IRS) were made using fluorenylmethyloxycarbonyl-based solid-phase peptide synthesis (Fmoc SPPS) in place of traditional bacterial expression systems (Drew et al., 2019). This method produced a high yield of proteins with intrinsic α-helical secondary structure and enabled the study of their biophysical properties in 1,2-dimyristoyl-sn-glycero-3-phosphocholine proteoliposomes to simulate a native-like environment. In addition, Fmoc SPPS was able to integrate spin-labels in the sequence to enable analysis by electron paramagnetic resonance and nuclear magnetic resonance (Ahammad et al., 2019). Using continuous-wave electron paramagnetic resonance, the authors were able to determine the mobility of each domain to reveal the structural topology of proteins with respect to the lipid bilayer. ^31^P and ^2^H solid-state nuclear magnetic resonance spectroscopy revealed the hydrophobic core and phosphorus head group interactions for the lipid bilayer (Drew et al., 2020).

Artificial intelligence and machine learning have emerged as potential tools to predict protein structures where existing methods are challenging, such as holins. Notably, AlphaFold (Jumper et al., 2021), which uses neural networks and deep learning techniques can predict protein structures with high accuracy. AlphaFold was trained on structures in the Protein Data Bank (Berman et al., 2000) to predict the distributions of distances between the β-carbon atoms of pairs of residues of a given protein and construct the 3D structure of the protein without using templates (Senior et al., 2020).

**Figure 2:**
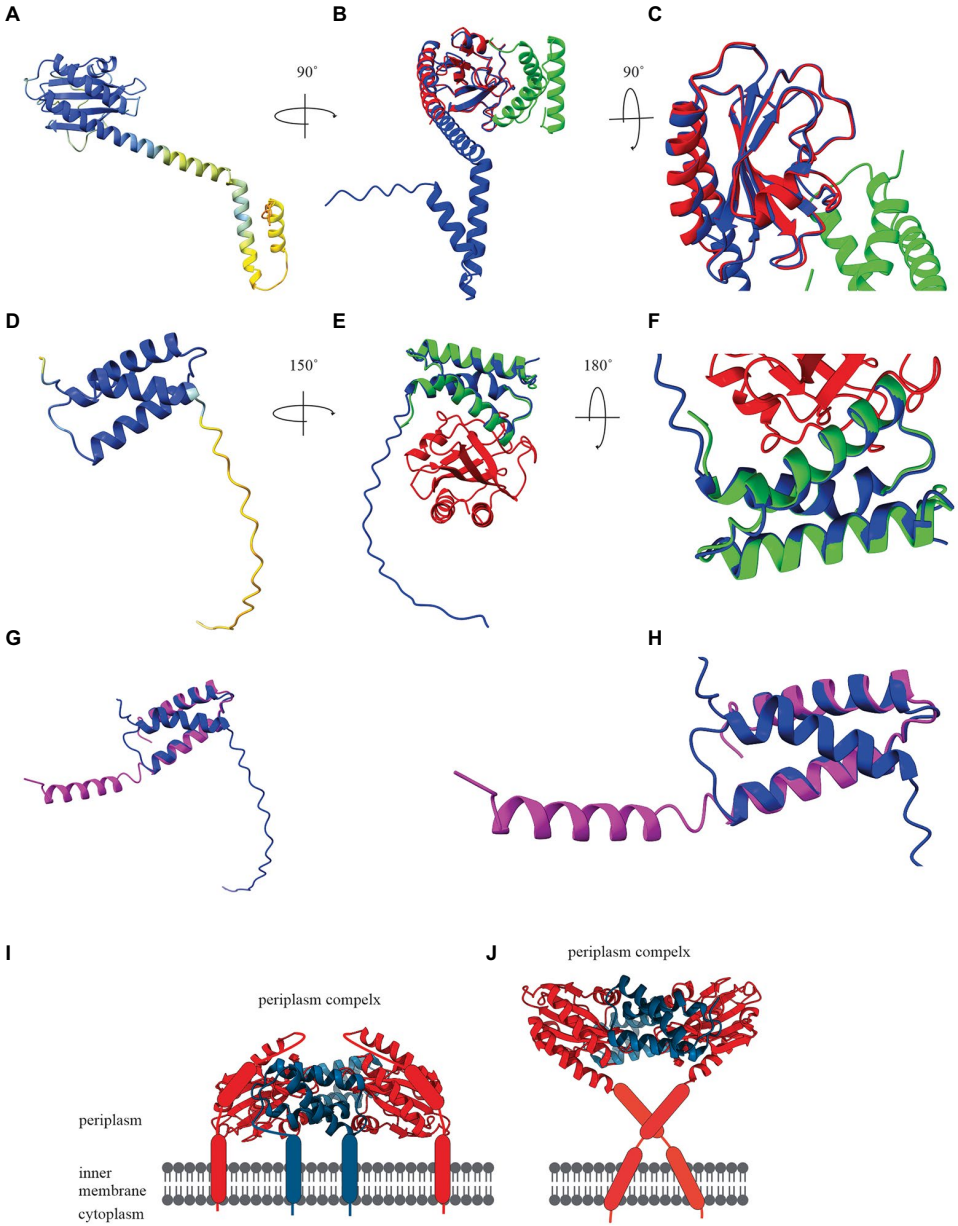
**(A)** AlphaFold Colab prediction of complete holin T structure. The colour of the predicted holin T structure represents the score of the predicted local distance difference test (pLDDT). **(B)** Structure comparisons of AlphaFold Colab predicted holin T (blue) and 6PSK(soluble domain of the resolved holin T and RI antiholin complex; RMSD = 0.688 angstroms). **(C)** An enlarged version of structure comparisons of AlphaFold Colab predicted holin T (blue) and 6PSK (red; soluble domain of holin T and green; soluble domain of RI antiholin). **(D)** AlphaFold Colab prediction of RI antiholin. The colour of the predicted RI antiholin structure represents the score of the predicted local distance difference test (pLDDT). **(E)** Structure comparisons of AlphaFold Colab predicted RI antiholin (blue) and 6PSK (soluble domain of the resolved holin T and RI antiholin complex; RMSD = 1.537 angstroms). **(F)** An enlarged version of structure comparisons of AlphaFold Colab predicted RI antiholin (blue) and 6PSK (red; soluble domain of holin T and green; soluble domain of RI antiholin). **(G)** Structure comparisons of AlphaFold Colab predicted RI antiholin (blue) and 6PSH (soluble domain of the resolved RI antiholin monomer; RMSD = 21.077 angstroms). **(H)** An enlarged version of structure comparisons of AlphaFold Colab predicted RI antiholin (blue) and 6PSH (purple). **(I)** RI–T complex model with RI SAR domain by Krieger et al. (2020). **(J)** RI–T complex model with cleavable signal peptide (new model) by Mehner-Breitfeld et al. (2021).

To demonstrate the potential of artificial intelligence and machine learning to determine a holin structure, we constructed the bacteriophage T4 holin T and antiholin RI structures using AlphaFold Colab under default settings (Figures 2A,D). AlphaFold Colab is a simplified version of AlphaFold that does not use homologous structures to construct structures (Jumper et al., 2021). We chose T4 holin T and antiholin RI as the crystal structures of the soluble periplasmic domains were available in the PDB (Krieger et al., 2020) and could be used to validate the predicted structures. The two main entries for the periplasmic domain T4 holin structures are holin T-antiholin RI complex (6PSK, 6PXE, and 6PX4) and free antiholin RI (6PSH; Krieger et al., 2020). Our analysis showed that the AlphaFold predicted structures of the soluble part of holin T and antiholin RI both aligned closely to the PDB structure of holin T-antiholin RI complex (6PSK; Figures 2B,C,E,F). On the contrary, the AlphaFold predicted antiholin RI structure did not match the PDB structure of the free antiholin RI (6PSH; Figures 2G,H). However, the PDB antiholin RI structure in the holin T-antiholin RI complex (6PSK) is more accurate than the PDB free antiholin RI (6PSH), as the latter crystallizes as a domain-swapped homo-tetramer (Krieger et al., 2020). Thus, AlphaFold predicted T4 holin T and antiholin RI structures could be useful predictors for their complete structures, including transmembrane domains.

Initially, it was thought that soluble periplasmic antiholin RI harboured a signal-arrest-release domain (Krieger et al., 2020). A model of the holin T-antiholin RI complex was made by Krieger et al. (2020) using this data (Figure 2I; Krieger et al., 2020). However, more recently, it was reported that RI has a cleavable signal peptide, not a SAR domain, and a new model of the RI–T complex was developed using the Chiron server (Figure 2J; Ramachandran et al., 2011; Mehner-Breitfeld et al., 2021). Surprisingly, the structure of holin T in the new model is a good match to the AlphaFold structure we predicted for holin T. Therefore, AlphaFold may be a good tool to determine holin and spanin structure and validate experimental structural data.

## Potential applications for membrane disrupting proteins

Bacteriophage encoded lysis proteins are being developed for a variety of applications across the medical, food, biotechnological, and pharmaceutical sectors. However, it is noteworthy that applications of spanins are not as widely reported as that of endolysins or holins. This may be as the cell wall degradation caused by the holin-endolysin system is sufficient alone to cause lysis in the industrial environment. For example, osmotic shock, shearing forces, or high temperatures could be sufficient to execute the function of the spanin (Young et al., 1979; Casjens et al., 1989; Berry et al., 2013). However, Rajaure et al. (2015) have shown the potential for using spanins to deliver drugs and biochemicals into cells by membrane fusion (Rajaure et al., 2015). Although the suitability of spanin for drug delivery fusion studies has not been validated previously, a similar fusion mechanism has shown potential to engineer exosome–liposome and live cells-liposomes hybrids (Sato et al., 2016; Sun et al., 2018).

In the medical sector, there is high demand for alternative treatments, such as holin and endolysin therapies, where their use is less likely to develop resistance compared to conventional antibiotics (Hermoso et al., 2007). Holins have also shown promise as enhancers for endolysin therapy. A synergistic effect was reported between holin and endolysin isolated from bacteriophage SMP in treating 12 bacterial strains, including strains of *Staphylococcus aureus*, *Bacillus subtilis*, and *Salmonella enterica* whereas holin alone lysed only two strains of *S. aureus* and *B. subtilis (*Shi et al., 2012*)*. A recent study showed that fused holin-endolysin protein (holin fused at the N terminus of endolysin) enhances catalytic activity against a broad range of multi-drug-resistant gram-negative and gram-positive bacterial pathogens (Basit et al., 2021). Holins could also be exploited to deliver targeted therapies to human cells. The manipulation of lysis time *via* holins in a suicidal strain of *Listeria monocytogenes* was shown to have the potential to deliver proteins or nucleic acids to human intestinal epithelial cells (Kuo et al., 2009). Another study demonstrated the ability of lambda holin to kill eukaryotic tumour cells including human mammary and cervix carcinoma cell lines *in vitro* and human embryonic kidney cell line HEK293 *in vivo*. It was thought that holins oligomerise in the membranes of cell organelles to kill tumour cells. This is due to the similarities of the eukaryotic organelle endomembrane to their prokaryotic endosymbiont progenitors (Agu et al., 2006).

Further studies need to be undertaken prior to starting clinical trials with holins and spanins. Protein properties such as conformations, activity and stability can be affected by pH, proteases or peptidases, and macrophage activity *in-vivo* (Uchenna Agu et al., 2001; Bruno et al., 2013; Saravanan et al., 2013). At physiological salt conditions, the electrostatic interactions between holins and the bacterial cell membrane may be less effective. Additionally, non-specific interactions between holins and other molecules (e.g., albumin, apolipoprotein A-I) are likely to be formed in the presence of human serum and plasma (Wang et al., 1998; Sivertsen et al., 2014; Mohamed et al., 2016; Starr et al., 2016). These interactions may reduce the treatment efficacy. Furthermore, hemolytic or cytotoxic effects need to be investigated to ensure the proteins do not localize in eukaryotic cell membranes and to understand the risk of releasing endotoxins when bacteria are lysed (Ozsoy et al., 2009; Laverty and Gilmore, 2014; Murray et al., 2021). Finally, the production cost and scalability of these proteins should be established as they may exceed that of traditional antibiotics (Boto et al., 2018). However, protein engineering, delivery systems such as liposome encapsulation, hydrophobicity or electrostatic modification through peptide sequence modification may be useful tools to overcome these challenges.

In the food sector, these membrane disrupting proteins could be used as effective food preservatives by preventing microbial growth. *E. coli* cell lysates containing an overexpressed holin-like protein Tmp1 inhibited the growth of gram-positive foodborne pathogens such as *B. subtilis* (Rajesh et al., 2011). Another study conducted on *L. monocytogenes*, an opportunistic foodborne pathogen responsible for listeriosis and a critical threat to public health, showed the potential of using HolGH15 holin as an antimicrobial agent (Song et al., 2021). Interestingly, HolGH15 decreased 10^6^ CFU mL^−1^ of *L. monocytogenes* to an undetectable level at 4°C, which is important as *L. monocytogenes* is one of the few pathogens able to grow at food refrigeration temperatures. Several studies suggest bacteriophage lysis proteins can be used as effective preservatives for a wide range of food items including fish, milk, cheese, eggs, and poultry (de Ruyter et al., 1997; Mayer et al., 2010; Han et al., 2014; Song et al., 2021).

There are also industrial applications for bacteriophage lysis proteins. For example, programmed autolysis of genetically engineered cyanobacteria by holin and endolysin induction with Ni^2+^ ions led to the effective release of hydrocarbons from the cells in biofuel production (Liu and Curtiss, 2009). Furthermore, it has been demonstrated that T4 holins and endolysins cloned in cyanobacterial cells can be engineered to be induced by green light to harvest biofuels (Miyake et al., 2014). In addition to biofuels, the holin-endolysin system has also been proposed to aid the release of several other valuable cytoplasmic biomaterials from microbes such as drugs, fatty acids, and nucleic acids efficiently and inexpensively (Gao et al., 2013).

## Perspectives and conclusions

Holins and spanins are a diverse group of bacteriophage-encoded bacterial membrane proteins and the diversity of these membrane disrupting proteins provides a fertile ground for developing novel antibacterial applications across many sectors.

Currently, most of the biomolecular, functional, and structural studies of holins and spanins are examined using *E. coli* expression systems, but this is challenging due to toxicity. Recent advances in protein synthesis and artificial intelligence (AI) and machine learning (ML) approaches are helping to overcome this challenge. However, our understanding of holins and spanins largely originates from studies in ionic buffers and detergents and so structural and dynamic information for these proteins in their native environment are not well understood. The use of cell-free systems such as nanodiscs, micelles, or giant unilamellar vesicles (GUVs; Gessesse et al., 2018; Novikova et al., 2018) could be a useful future approach to express and analyse these toxic membrane proteins.

Using native mass spectrometry for analysing membrane proteins has advantages compared to X-ray crystallography or NMR such as speed, capability to deal with heterogeneous samples, and lower limits of detection. Advancements in ion sources used for mass spectrometry, for instance, nano-electrospray ionization (nESI) and laser-induced liquid bead ion desorption (LILBID), shows promising results for analysing membrane proteins in their native form (Peetz et al., 2018). Similarly, hydrogen deuterium exchange (HDX), fast photochemical oxidation of proteins (FPOP), and cross-linking mass spectrometry (XLMS) could be valuable new tools to determine membrane protein characteristics such as protein–protein or protein-lipid interactions, aggregations, determining binding affinity, hidden conformations, and structure elucidation (Li et al., 2016; Lu et al., 2016; Frieden et al., 2017; Wang et al., 2017; Van et al., 2020). As such these techniques could be used to uncover the topology, dynamic functions and oligomerisation of holins and spanins more rapidly and cost-effectively.

Revealing the functional and dynamic mechanisms of holins and spanins would help develop medical and commercial applications. For example, optimization of the lysis clock mechanism of holin endolysin engineered bacterial cells would be economically beneficial for some applications such as bio-fermentation, biofuel and bio-based chemicals. Exogenous applications could be challenging as aggregates of 3 × 10^3^ canonical holins (Zagotta and Wilson, 1990; Chang et al., 1995) or 7 × 10^3^ pinholins (Pang et al., 2009, 2013) per cell are needed for membrane disruption. Moreover, triggering is regulated by allele type, antiholin ratio and more importantly biochemical environment of the cytoplasm and periplasm. Surprisingly, several studies have shown that the synergistic effect between endolysins and holins results in beneficial outcomes for treating multidrug-resistant bacteria and carcinogenic cells (Agu et al., 2006; Shi et al., 2012). These findings suggest the potential for wider applications of engineering the holin-like protein family, such as Bax and Bak, to selectively permeabilize eukaryotic cell membranes. There is a large knowledge gap in the exogenic application potential of these proteins which needs to be addressed for their full potential to be realised.

There are, however, many questions still to be addressed. For example:

- On the lysis clock: Can the triggering time be predicted or optimized precisely? How can the role of antiholin function in triggering be described in antiholin free phage life cycle? Do antiholin free bacteriophages have non-dual-start antiholins?
- Regarding oligomerization: What are the key biophysical properties of holins that drive the formation of death-raft or pinholin oligomerisation?
- On exogenic membrane permeabilization: Do exogenic treatment effects primarily arise from inhibition, toxicity, or outer membrane permeabilization?
- Biophysical characterization: Which techniques are suitable to handle low quantity, heterogenic samples to characterize these proteins? How to create an environment to mimic the proton motive force in planar lipid bilayer systems?
- Toxicity and stability studies: Can holins or spanins oligomerize in mammalian organelle cell membranes? What would be the autoimmune reaction in mammalian cells? What is the resistance to proteases?
- Scaling up: What would be the best strategy to maximize the holin and spanin yield? What is the best way to manage leaky expressions?

To summarize, holins and spanins have co-evolved to disrupt the bacterial cell membrane. The elegant membrane disruption mechanisms of these proteins have only recently begun to be fully understood and advances in analytical techniques have promise to further advance our understanding. Studying the biophysical and mechanistic properties of these membrane disruptors will answer some fundamental questions in cell biology and open new avenues for their biotechnological use.

## Author contributions

GA wrote the manuscript, with comments from ML and SM. CB and RD edited the manuscript. All authors contributed to the article and approved the submitted version.

## Funding

This study was supported by funding from the University of Canterbury Biomolcular Interaction Centre and the Institute of Environmental Science and Research (ESR). GA is supported by a ESR Vision PhD Scholarship.

## Conflict of interest

The authors declare that the research was conducted in the absence of any commercial or financial relationships that could be construed as a potential conflict of interest.

## Publisher’s note

All claims expressed in this article are solely those of the authors and do not necessarily represent those of their affiliated organizations, or those of the publisher, the editors and the reviewers. Any product that may be evaluated in this article, or claim that may be made by its manufacturer, is not guaranteed or endorsed by the publisher.
